# Living donor liver transplantation for neonatal hemochromatosis using non-anatomically resected segments II and III: a case report

**DOI:** 10.1186/1752-1947-4-372

**Published:** 2010-11-19

**Authors:** Amit Sharma, Adrian H Cotterell, Daniel G Maluf, Marc P Posner, Robert A Fisher

**Affiliations:** 1 Department of Surgery, Hume-Lee Transplant Center, Virginia Commonwealth University, PO Box 980057, Richmond, Virginia 23298-0057, USA

## Abstract

**Introduction:**

Neonatal hemochromatosis is the most common cause of liver failure and liver transplantation in the newborn. The size of the infant determines the liver volume that can be transplanted safely without incurring complications arising from a large graft. Transplantation of monosegments II or III is a standard method for the newborns with liver failure.

**Case presentation:**

A three-week old African-American male neonate was diagnosed with acute liver failure secondary to neonatal hemochromatosis. Living-related liver transplantation was considered after the failure of intensive medical therapy. Intra-operatively a non-anatomical resection and transplantation of segments II and III was performed successfully. The boy is growing normally two years after the transplantation.

**Conclusion:**

Non-anatomical resection and transplantation of liver segments II and III is preferred to the transplantation of anatomically resected monosegements, especially when the left lobe is thin and flat. It allows the use of a reduced-size donor liver with intact hilar structures and outflow veins. In an emergency, living-related liver transplantation should be offered to infants with liver failure secondary to neonatal hemochromatosis who fail to respond to medical treatment.

## Introduction

Neonatal hemochromatosis (NH), although rare, is the most common cause of liver failure and liver transplantation in neonates. Liver transplantation is the main therapy for infants who fail to respond to medical treatment [1]. Liver transplantation using either mono-segment II or III [2,3] is a technically challenging option that is especially beneficial for small infants in whom a left lateral segment [4] is large-for-size. We report that non-anatomical resection and transplantation of segments II and III may be a simpler, yet effective, surgical option for neonates with liver failure.

## Case presentation

A three-week old African-American male newborn, weighing 2.5 kg, was admitted to our unit with jaundice, abdominal distension and hepatomegaly. The pregnancy had been uncomplicated and there was no family history of metabolic or liver disease.

Laboratory studies for liver failure revealed a total serum bilirubin of 22.5 mg/dL, an international normalized ratio 4.9, aspartate aminotransferase 45 U/L and alanine aminotransferase 23 U/L. The boy had a serum iron of 157 μg/dL (normal 30-165 μg/dL), serum ferritin 994 ng/mL (normal 30-330 ng/mL), serum transferrin 103 mg/dL (normal 215-380 mg/dL) and transferrin saturation 109% (range 16%-60%). He had an elevated alpha-fetoprotein level (3289 ng/mL). Investigations for infectious and inherited metabolic pathologies were negative. Magnetic resonance imaging (MRI) of the abdomen was suggestive of iron deposition in the liver, with conventional, patent arterial and venous anatomy. NH was suspected and confirmed by minor salivary gland biopsy from the lower lip.

Medical therapy consisting of anti-oxidants and chelation with desferroxamine was initiated. As there was a progressive worsening of the boy's condition on medical therapy, his mother volunteered to be a living liver donor. After a standard expedited two-day donor-workup, the related living donor liver transplantation was planned. Pre-operative MRI showed that the mother's left lateral segment volume was approximately 200 cc^3^. We therefore decided to do a left lateral resection with back-table monosegmentectomy followed by transplantation.

**Figure 1:**
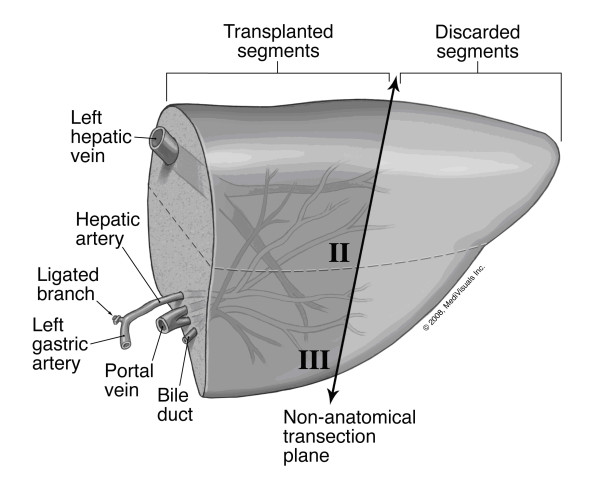
**Liver segments II and III with reconstructed hilar structures just before non-anatomical resection**. The donor had a left hepatic arterial branch arising from the left gastric artery and two left hepatic vein tributaries draining into the supra-hepatic vena cava. The final arrangement of the left portal vein, the reconstructed hepatic veins, replaced left hepatic artery arising from the left gastric artery and the bile duct is demonstrated here. On the back-table, transection was carried through segments II and III along a non-anatomical plane (thick black line) using an endovascular stapling device.

Intra-operatively, after isolation of the mother's left hepatic artery, hepatic duct and portal branch, the hepatic parenchyma of segment IV was transected 5 mm to the right of the falciform ligament without blood inflow occlusion or graft manipulation. The segment II and III ducts united, just lateral to the umbilical portion of the left portal vein and the segment IV duct, then joined medial to umbilical portion. This confluence of segment II and III ducts was divided and used for anastomosis in the recipient. This was a thin 'pancake' left lateral segment that was transected using ultrasound guidance. On the back-table, a 2.5 mm endostapler with two, triple-staggered rows of titanium staples (Autosuture™GIA™UNIVERSAL stapler, US Surgical, Division of Tyco Healthcare Group LP, CT, USA) was used to staple and divide across the mid-portion of the left lateral segment just to the left of the secondary portal vasculature branching (Figure 1). The final graft consisted of the confluence of segment II and III bile ducts, left portal vein, left hepatic artery arising from left gastric artery and two in-proximity left hepatic veins, joined as one. This non-anatomically resected portion of the lateral segment was used for transplantation in standard piggy-back fashion with Roux-en-Y jejuno-biliary anastomosis. The discarded part was used for hepatocyte isolation [5]. Heparin and aspirin were used in the first post-operative week to prevent vascular thrombosis. The patient was re-explored in the first week for clot evacuation around the transplanted liver segments, with no active bleeding on the cut surface or the stapled edge. Patient was discharged home after three weeks and continues to do well two years post-transplant.

## Discussion

Neonatal hemochromatosis is a syndrome with an aggressive course and a poor prognosis. The etiopathogenesis is not very clear although siderosis resulting from infections, genetics and auto-immunity may play a role [6]. The pregnancy may be complicated by intrauterine growth restriction, oligohydramnios or still birth. The neonate may present with signs of hepatic insufficiency within hours of birth. Abnormal laboratory parameters include: decreased transferrin; ceruloplasmin; increased ferritin (non-specific, > 800 ng/mL); mixed hyperbilirubinaemia; low aminotransferases; low factors V and VII (< 10% of normal); thrombocytopenia, anaemia and increased alpha-fetoprotein (> 200 ng/mL). Hepatic and extra-hepatic siderosis with reticuloendothelum sparing is diagnostic of NH. Lower lip biopsy is safe and convenient for documenting siderosis in minor salivary glands. MRI is used to support the diagnosis of NH and is characterised by low signal intensity on T2 weighted liver images [7]. Since NH recurs in 75-80% of siblings, the parents should be discouraged from having any further pregnancies. Gestational high dose intravenous immunoglobulin administered to the mother, from 18 weeks to birth, appears to decrease the lethality of recurrent neonatal hemochromatosis [8]. Medical therapy with desferrioxamine and antioxidant cocktail (N-acetylcysteine, vitamin E, prostaglandin E1 and selenium), although not highly successful, are still used to treat neonates. Liver transplantation is considered to be the treatment of choice for infants not responding to medical therapy. Early medical therapy results in a 10%-20% survival rate while long-term survival after liver transplantation may range from 50% to 66% [1].

Liver transplantation using either monosegment II or III is a useful option for small infants in whom a whole left lateral segment is large-for-size [4]. Monosegment transplantation is mostly used for infants with a calculated graft-to-recipient weight ratio of less than or equal to 4.0% when using the left lateral segment. Splitting of the left lateral segment can be been done either *in situ *in the donor or on the back-table. Despite these surgical innovations, neonatal liver transplantation still poses challenges because of the size of the recipients who usually weigh less than 10 kg [9]. Depending on the donor size, even the transplanted monosegment may be large-for-size and make graft placement technically difficult and may lead to post-operative complications [10]. More importantly, the use of a segment II or III may result in a smaller diameter bile duct that may be more prone to strictures and leaks as the liver regenerates [1]. In our report, the left lateral segment was split along a non-anatomical plane and the confluence of segments II and III bile ducts provided us with a larger caliber (4 mm) duct in the donor segments. The caliber of the hepatic and the portal veins used for anastomosis were the same as when using a complete left lateral segment. The use of a stapling device made this division technically easier and more efficient. However, the stapled edge may be prone to bleeding after reperfusion. This can be minimized by selecting thin and flat left lateral segments for stapling. This case also demonstrates that emergent living-related liver transplantation is a viable option for neonates with acute liver failure who may not survive the time spent on the waiting list for a whole or a split-liver from a deceased donor.

## Conclusion

Urgent living-related liver transplantation should be offered to infants with acute liver failure secondary to neonatal hemochromatosis who are non-responsive to medical therapy. The left lateral segment can be reduced in size, especially when it is flat (like a pancake), by splitting it along a non-anatomical plane. This simple technique allows the use of the confluence of donor segment II and III bile ducts that are less prone to stricturing due to their larger caliber. However, this advantage may be lost in cases where the segment II and III bile ducts join separately, medial to the umbilical portion of the portal vein.

## Abbreviations

IUGR: intrauterine growth restriction; MRI: magnetic resonance imaging; NH: neonatal hemochromatosis.

## Competing interests

The authors declare that they have no competing interests.

## Authors' contributions

AS collected data, designed and wrote the manuscript. AHC was a major contributor to the manuscript. DGM assisted in the critical revisions of the manuscript. MPP reanalyzed the surgical facts and provided comments on the critical intellectual content of the manuscript. RAF helped to conceive, critically revise and write the manuscript. All authors read and approved the final manuscript.

## Consent

Written informed consent was obtained from the patient's mother for publication of this case report and any accompanying images. A copy of the written consent is available for review by the Editor-in-Chief of this journal.
